# Nanoscale optical and electrical characterization of horizontally aligned single-walled carbon nanotubes

**DOI:** 10.1186/1556-276X-7-682

**Published:** 2012-12-21

**Authors:** Raul D Rodriguez, Marius Toader, Sascha Hermann, Evgeniya Sheremet, Susanne Müller, Ovidiu D Gordan, Haibo Yu, Stefan E Schulz, Michael Hietschold, Dietrich RT Zahn

**Affiliations:** 1 Semiconductor Physics, Chemnitz University of Technology, Chemnitz, D-09107, Germany; 2 Solid Surfaces Analysis Group, Chemnitz University of Technology, Chemnitz, D-09107, Germany; 3 Center for Microtechnologies (ZfM), Chemnitz University of Technology, Chemnitz, D-09107, Germany; 4 Fraunhofer Institute for Electronic Nano Systems (ENAS), Chemnitz, 09126, Germany

**Keywords:** Single-walled carbon nanotubes, CNT transistor, Raman imaging, Current sensing AFM, Atomic force microscopy

## Abstract

During the recent years, a significant amount of research has been performed on single-walled carbon nanotubes (SWCNTs) as a channel material in thin-film transistors (Pham et al. *IEEE Trans Nanotechnol* 11:44–50, 2012). This has prompted the application of advanced characterization techniques based on combined atomic force microscopy (AFM) and Raman spectroscopy studies (Mureau et al. *Electrophoresis* 29:2266–2271, 2008). In this context, we use confocal Raman microscopy and current sensing atomic force microscopy (CS-AFM) to study phonons and the electronic transport in semiconducting SWCNTs, which were aligned between palladium electrodes using dielectrophoresis (Kuzyk *Electrophoresis* 32:2307–2313, 2011). Raman imaging was performed in the region around the electrodes on the suspended CNTs using several laser excitation wavelengths. Analysis of the G^+^/G^−^ splitting in the Raman spectra (Sgobba and Guldi *Chem Soc Rev* 38:165–184, 2009) shows CNT diameters of 2.5 ± 0.3 nm. Neither surface modification nor increase in defect density or stress at the CNT-electrode contact could be detected, but rather a shift in G^+^ and G^−^ peak positions in regions with high CNT density between the electrodes. Simultaneous topographical and electrical characterization of the CNT transistor by CS-AFM confirms the presence of CNT bundles having a stable electrical contact with the transistor electrodes. For a similar load force, reproducible current–voltage (*I*/*V*) curves for the same CNT regions verify the stability of the electrical contact between the nanotube and the electrodes as well as the nanotube and the AFM tip over different experimental sessions using different AFM tips. Strong variations observed in the *I*/*V* response at different regions of the CNT transistor are discussed.

## Background

Due to their exceptional properties, carbon nanotubes (CNT) have been the focus of intense research in several fields from spintronics to biosensing [1,2]. Moreover, recently, CNTs are being explored as active materials for the next generation of sensing devices, solar cells, field effect transistors (FET), and nanoelectronics [3-6]. Pioneered by the work of Tans et al. [7], one of the promises of nanotechnology using carbon nanotubes concerns the development of faster, more power-efficient and smaller electronic devices [8]. However, the realization and mass production of CNT electronics have remained elusive so far. It is a complex situation since the large-scale integration of carbon nanotubes into current silicon technology is still under development. One of the main challenges concerns the selective deposition of carbon nanotubes on predefined positions of a circuit such as across a channel in a FET device. In this regard, dielectrophoresis offers a good advantage since it is possible to control the position and alignment of the CNTs along electrodes in an integrated circuit [9]. In addition, dielectrophoresis technology can be made compatible with mass-production processes while allowing deposition directly from CNTs dispersed in liquid [10,11]. In this work, we undertake the study of semiconducting single-walled CNTs that have been aligned and deposited along two pre-structured palladium electrodes with a channel separation of 2 μm. Using jointly Raman spectroscopy imaging and current sensing AFM (CS-AFM), we aim at investigating the properties of dielectrophoresis-deposited carbon nanotubes in order to find out whether or not the defect concentration in carbon nanotubes increases at the CNT/electrode interface, evaluating at the nanoscale level the quality of the electrical contact between the nanotubes and the electrodes (Ohmic or not) and verifying that a good alignment can be achieved along the channel. In addition to the defect concentration obtained from the intensity ratio of the D/G band, from Raman spectroscopy, the CNT diameter was estimated using the splitting of the G^−^ and G^+^ peaks [12].

## Methods

**Figure 1:**
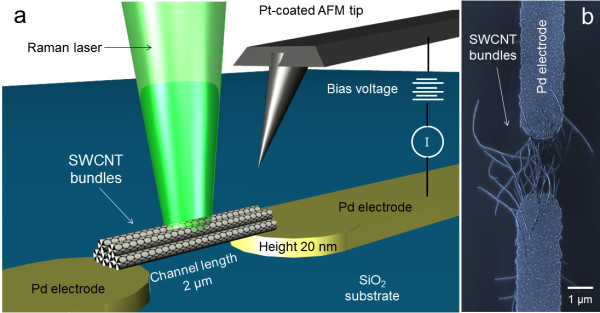
**CNT bundles aligned along the channel made by two palladium electrodes on a SiO_2_ surface (a).** Raman measurements were performed in the backscattering geometry. Scanning electron micrograph of the CNTs between the electrodes (**b**).

A CNT transistor structure was prepared using p-type silicon with (100) crystal orientation covered with a 1,000-nm thick SiO_2_ dielectric layer. Pd (10 nm)/Al (10 nm) electrodes were prepared by sequential dry and wet etching procedures. The design of the CNT device is shown in a scheme in Figure 1a, while in Figure 1b, a scanning electron micrograph of the actual device is shown. Subsequently, purified and type-selected CNTs (98% semiconducting provided by NanoIntegris Inc., CA, USA), dispersed in deionized water containing 0.2 wt.% of sodium dodecyl sulfate, were deposited and aligned between the electrodes by dielectrophoresis [13].

CS-AFM data were recorded with a 5500 AFM from Agilent Technologies (CA, USA) using Ti/Pt-coated AFM probes (tip radius < 40 nm) with a spring constant of approximately 0.12 N/m.

Raman measurements were performed in the backscattering geometry within the spectral range of 1,100 to 2,800 cm^−1^, which includes the first and the second order bands using the 488 and 514.5 nm lines of an Ar^+^ laser and the 632.8 nm line of a HeNe laser. The Raman spectrometer is a LabRam HR800 (HORIBA Scientific, Villeneuve d’Ascq, France) with an optical microscope Olympus BX40 (Olympus Europa Holding GmbH, Hamburg, Germany). A 100× objective (N.A. 0.9) was used to illuminate the sample and to collect the Raman signal with a diffraction limited resolution of *λ* / (2 N.A.) ≈ 286 nm (*λ* = 514.5 nm). A liquid nitrogen-cooled back-illuminated charge-coupled device (CCD) was employed for the detection of the Raman signal using a diffraction grating of 600 l/mm yielding a spectral resolution of 4 cm^−1^. The laser power was limited to the range of 0.5 to 2 mW in order to prevent sample damage. Full Raman spectra were acquired with a Raman imaging stage with a step size of 500 nm.

## Results and discussion

**Figure 2:**
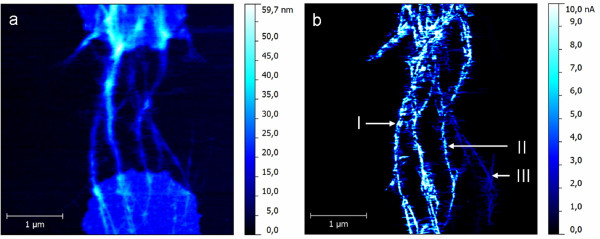
**Topography (a) and current map (b) with +2 mV sample bias.** The regions I, II, and III are discussed in the main text.

In Figure 2a, a classical topographical AFM image and the corresponding current map are displayed. The images were simultaneously recorded in contact mode, which is known to be the most destructive AFM scanning mode, but here required in order to obtain the corresponding current response. However, upon multiple scanning frames, the CNTs-FET structure remains unchanged emphasizing good contact stability at the CNT/metal electrode interface. According to the topography, the CNTs form bundles, parallel agglomerates, which are long enough to bridge the gap and, therefore, to interconnect the two metal electrodes. Using +2 mV sample bias, the corresponding current map is displayed within Figure 2b. While the tubes give a constant current response along the entire length, the metal electrodes could not be observed in the current map. This is most probably due to an insulating layer formed at the corresponding surface as a result of residual photoresist [14]. Since a current response along the CNTs could be observed, it can be assumed that the electrical contact is established between the CNTs and the two metal electrodes. This might be possible if the CNT/electrode contact is buried below this insulating layer, and therefore, a corresponding current response can be detected along the CNTs. Moreover, platinum (the coating material of the AFM probes) is well known to have a good adhesion to CNTs, and consequently, a good electric contact is expected.

**Figure 3:**
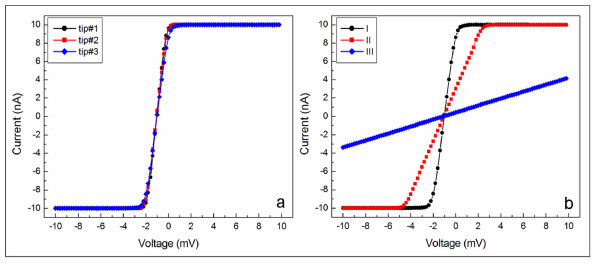
**Current–voltage characteristics obtained.** The same CNT (I) using different AFM probes (**a**); different CNTs using the same AFM probe (tip #3) (**b**).

For a better insight into the electric behavior of the CNTs, current–voltage spectroscopy was used. However, for a comprehensive study, the corresponding reproducibility of the *IV* spectra has to be checked. Therefore, for the marked CNT (I), the same kind of AFM probes were used in successive working days. Multiple *IV* sets averaged over 10 spectra were recorded for the same location. One hundred points and 2-s acquisition time were used for each individual spectrum. Spectra (40, 60, and 120) were recorded using the tips #1, #2, and #3, respectively (see Table 1). The corresponding average spectra are displayed in Figure 3a. Regardless of the used AFM probe, the current–voltage characteristics are highly reproducible. Between the two saturation regimes, which represent the current limitation of our device (±10 nA), a linear *IV* dependence was observed. This emphasizes a good Ohmic conduction at the CNT/metal interface. The values for the estimated resistance are included in Table 1, in good agreement with a previous transport study in the SWCNT networks [15]. It should be pointed out that these values contain a signature arising from multiple contacts namely, the AFM tip/CNT, CNT/metal electrode, and metal electrode/tungsten metallic wire (used to contact the sample).

**Table 1:** CNT resistance values estimated from CS-AFM

	**Tip** #**1**	**Tip** #**2**	**Tip** #**3**		
CNT	I			II	III
Resistance (kΩ)	85	96	103	349	2,630

Regions I, II, and II are shown in Figure 2.

While the first and the last contributions are constant and negligible, the contact between the CNT and the metal electrode is of great importance. As can be observed from the bottom part of the topography image in Figure 2a, the contact (which equals the interface path between the CNTs and the metal surface) is different from bundle to bundle. Accordingly, there seems to be a good correlation with the current response. For the marked CNTs, the detected current passing through is gradually decreasing relative to the contact. This is most probably due to different quality of the contact and, therefore, different values for the contact resistance. The average spectra for the investigated CNTs recorded using the same AFM probe are shown within Figure 3b, while the corresponding estimated resistance values are included in Table 1.

**Figure 4:**
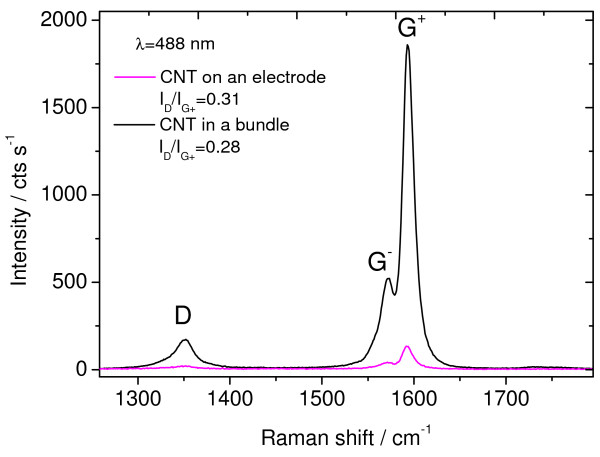
**Raman spectra of the CNT-FET structure.** At the channels (black curve) and at the electrodes (pink curve) using an excitation wavelength of 488 nm. The main bands characteristic of carbon nanostructures are visible: D band at 1,351 cm^−1^, G^−^ at 1,571 cm^−1^, and G^+^ at 1,593 cm^−1^.

The quality of the CNTs was probed by Raman spectroscopy. As shown in Figure 4, the Raman spectrum of the CNTs displays characteristic peaks in the spectral range of 1,200 to 1,800 cm^−1^. The G feature is a characteristic peak appearing around 1,582 cm^−1^ which is universal to all carbon structures having *sp*^2^ hybridization [16]. The leftmost band, around 1,351 cm^−1^ (for *λ* = 488 nm) is known as the D band (defect-induced), and it requires a structural defect to be active in the otherwise perfect honeycomb carbon lattice. Due to the curvature of SWCNTs, in contrast to the perfect honeycomb lattice of graphite, the G band splits into the G^+^ and G^−^ bands centered around 1,571 and 1,593 cm^−1^, respectively, as shown in Figure 4. The shape of the G^−^ band is characteristic for semiconducting (Lorentzian shape) or metallic (Breit-Wigner-Fano shape) nanotubes; for metallic CNT, this band is quite broad and as intense as the G^+^. The G^+^ band is sensitive to doping (blue shift for acceptors and red shift for donors) [17]. The G band splitting becomes less pronounced as the CNT diameter increases and disappears for large CNT radii or for the case of multi-walled CNTs. In such case, the Raman peak has a similar lineshape like the G band observed in graphite and graphene. The ratio between the intensities of D and G bands is correlated with the amount of defects in graphitic materials, and it can be related to the average distance between defects using the Tuinstra-Koenig relation [18] or a recent phenomenological model proposed by Lucchese et al. [19].

**Figure 5:**
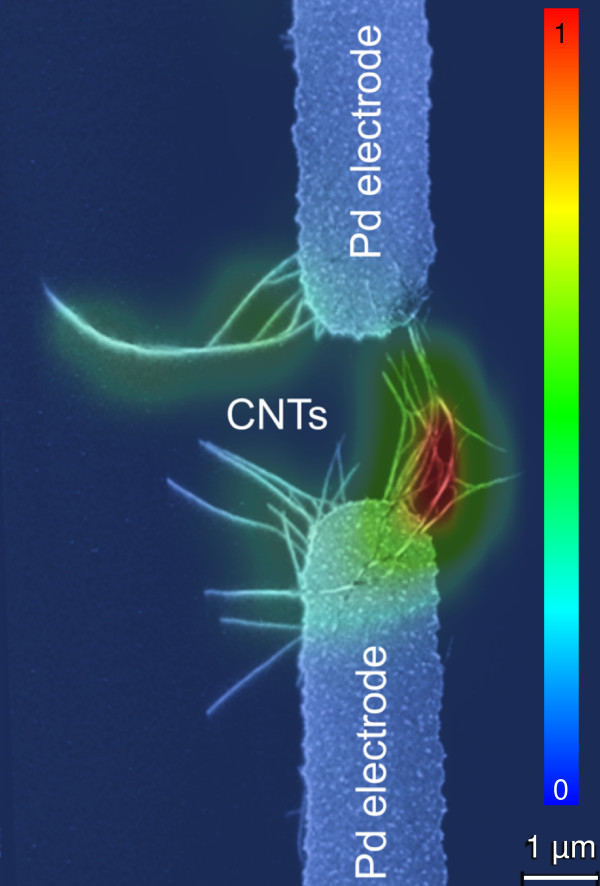
**Raman map superimposed to a scanning electron micrograph of the FET.** The color code indicates the intensity of the G^+^ band using an excitation wavelength of 632.8 nm.

**Figure 6:**
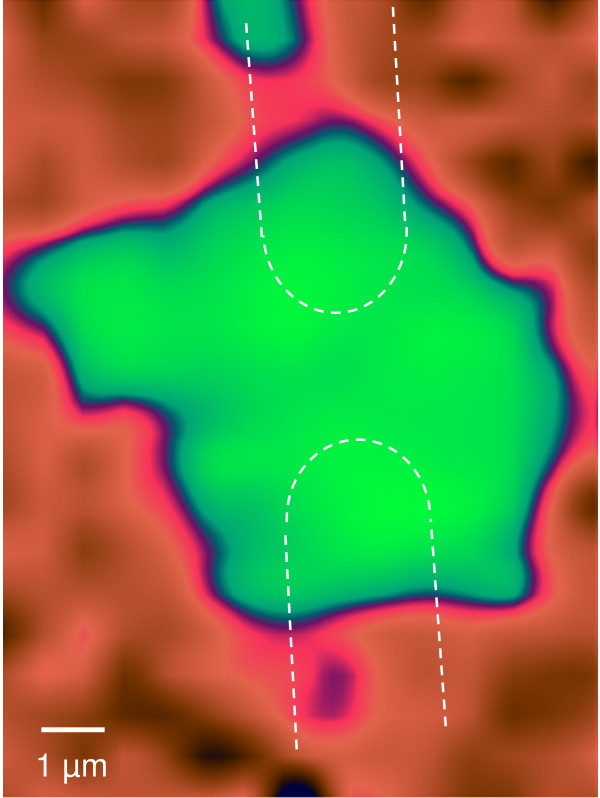
**Map of the D/G^+^ peak intensity ratio of the FET.** The green color around the two electrodes sketched by dashed lines represents values of 0.31 ± 0.02. In red and dark color, the intensity ratio is not defined due to the absence of Raman signal in those regions. No particular increase in defect concentration is observed at the CNT/electrode interface.

Acquiring Raman spectra across a sample in a point-wise form allows identifying sample heterogeneities coming from differences in physico-chemical properties made visible in the Raman spectra like in Figures 5 and 6. This research area, involving the two-dimensional mapping of structural properties using Raman spectroscopy, has been fueled by recent developments in coupling Raman with scanning probe techniques. Such coupling has given rise to the so-called tip-enhanced Raman spectroscopy. In this work, we focus only on micro-Raman imaging which gives a spatial resolution of roughly half the wavelength used for Raman excitation.

Avoiding metallic CNTs in a transistor is of great importance since few metallic carbon nanotubes can create a shortcut, compromising the transistor performance. Giving their clear different signature, in our Raman imaging results, metallic CNTs were not detected but only semiconducting ones [16]. It is possible that the 2% of metallic CNTs present in the original solution were burnt out during the dielectrophoresis deposition [9] or their amount is not sufficient to be detected. Due to the metallic nature of the Pd electrodes and their roughness, surface-enhanced Raman spectroscopy might appear in regions where the CNT was in direct contact with the electrodes. However, we did not find any visible SERS effect which could be explained by the possible presence of residual photoresist that has also hidden the metallic electrode from the conductive AFM probe evidenced in CS-AFM as discussed above.

The assessment of CNT diameter using Raman spectroscopy has been the subject of intense research, mainly based on the analysis of the radial breathing modes (RBM) and their frequency positions at different excitation energies using the so-called Kataura plot [16,17,20]. However, this method requires as many Raman excitation lines as possible using a tunable laser in order to determine resonance energies of the CNT related with optical transitions; in addition, the RBM band is very sensitive to the tube environment. For this task, the three laser lines used in this work were not enough. However, G^−^/ G^+^ modes being in-plane vibrations are less sensitive to environmental changes [21]. Therefore, a rough estimation of the diameter (*d*) of CNTs deposited in the transistor was obtained by evaluating the splitting of the G^−^ and G^+^ bands following an empirical formula recently proposed by Telg et al. [12].

(1) $$\omega_{\mathrm{ph}}=a_{0}+\frac{a_{1}}{d}+\frac{a_{1}}{d^{2}}\text{,}$$

where *a*_0_ = 1,582 cm^−1^, *a*_1_ = −27, and *a*_2_ = 0 are parameters taken from Table 2 of reference [12] for the frequency shift *ω*_*ph*_ of the G^−^ observed in this work. Diameter estimations for different wavelengths are shown in Table 2. The discrepancy among estimations based on Raman data obtained with 632.8 nm excitation is a consequence of an artifact in the CCD detector for the spectral region in italics (etaloning effect).

**Table 2:** Summary of the peak positions and intensity ratios

***λ *(nm)**	**G**^**−**^**(cm**^**−1**^**);d(nm)**	**G**^**+**^**(cm**^**−1**^**)**	***I***_**D**_**/*I***_**G+**_
488	1,571 ± 1; 2.50	1,593 ± 1	0.28 (0.31)
514.5	1,572 ± 1; 2.75	1,593 ± 1	0.27 (0.30)
*632.8*	*1,567 ± 5; 1.83*	*1,592 ± 5*	*0.31 (0.31)*

The estimation of the CNT diameter was obtained using Equation 1 and the values of the G^−^ peak position. The values of the intensity ratios shown between parentheses were obtained from the Raman spectra of CNTs on the electrodes, while values outside parentheses were taken in between the electrodes.

The obtained local intensities of the G^+^ band are displayed in the Raman map shown in Figure 5. The *I*_D_/*I*_G_ ratio for CNT bundles between the electrodes and on the electrodes is shown in the mapping of Figure 6. The *I*_D_/*I*_G_ ratio appears similar for different excitation wavelengths having a value of 0.29 ± 0.02 for CNTs on the bundles between the electrodes and a *I*_D_/*I*_G_ ratio of 0.30 ± 0.01 for CNTs on the electrode. The shape of the three peaks (D, G^+^, and G^−^) does not change throughout the investigated region. Given that the Raman imaging shows a homogeneous CNT quality along the FET, differences in resistance observed by CS-AFM between different bundles can most certainly be attributed to the quality of the Pd electrode/CNT contact, and not to the CNT quality. A slightly higher defect concentration observed at the CNTs on the electrodes might come from welding of the CNT onto the Pd electrode during deposition, although such small difference in *I*_D_/*I*_G_ ratio is within the experimental error.

## Conclusions

Raman spectroscopy and imaging in addition to current sensing AFM were used in order to investigate a CNT-based device. Semiconducting single-walled CNTs were deposited and aligned using dielectrophoresis. The semiconducting character of the CNT bundles was proved by Raman spectroscopy, and the SWCNT diameter was determined to be 2.5 ± 0.3 nm. It is shown that an Ohmic contact between the palladium electrodes and the CNTs is realized using this fabrication method without any significant increase in defect density at the CNT/electrode contact.

## Competing interests

The authors declare having no competing interests.

## Authors’ contributions

RDR wrote the manuscript, coordinated between all the participants, contributed to the design of the study, and performed all the Raman imaging experiments and the data analysis. MT performed all the current sensing AFM experiments and the data analysis and wrote the section of CS-AFM. SH made all the CNT-FET devices and coordinated between all participants. ES contributed to the Raman spectroscopy and imaging experiments, data analysis, and read and improved the manuscript. SM participated in the AFM and Raman experiments and made significant corrections and improvements to the manuscript. ODG participated in the coordination and design of the experiments and read and corrected the manuscript. HY participated in the preparation of the CNT samples. SES, MH, and DRTZ participated in the conception of the project, coordinated among all the participants, and read and improved the manuscript. All authors read and approved the final manuscript.
